# Aspirin in people with dementia, long-term benefits, and harms: a systematic review

**DOI:** 10.1007/s00228-021-03089-x

**Published:** 2021-01-22

**Authors:** Katrina A. S. Davis, Delia Bishara, Mariam Molokhia, Christoph Mueller, Gayan Perera, Robert J. Stewart

**Affiliations:** 1 King’s College London Institute of Psychiatry Psychology and Neuroscience, London, UK; 2 South London and Maudsley NHS Foundation Trust, London, UK; 3 King’s College London Population Health Sciences, London, UK

**Keywords:** Aspirin, Dementia, Multimorbidity, Systematic review, Evidence-based medicine

## Abstract

**Purpose:**

People with dementia may have indications for aspirin prescription and clinicians are asked to balance the potential risks against benefits. This review examines the evidence for the risk and benefit of long-term aspirin use in people with dementia aged over 65 years, including randomised controlled trials and observational studies.

**Methods:**

We searched three databases for research published between 2007 and 2020. Each eligible article was assessed for risk of bias, and confidence in findings was rated using Grading of Recommendations Assessment, Development and Evaluation (GRADE).

**Results:**

Four papers met inclusion criteria: one randomised controlled trial, two cohort studies, and one with pooled data. All looked only at dementia of Alzheimer’s type, and none addressed myocardial or cerebral infarction as outcomes. Dementia progression was reported by two studies, with conflicting results. The trial found no significant effect of aspirin on mortality (odds ratio aspirin vs. no aspirin 1.07, 95% confidence interval 0.58–1.97) but found more events of severe bleeding with aspirin (OR aspirin vs. no aspirin 6.9, 1.5–31.2). An excess in intracranial haemorrhage in the aspirin group was judged plausible based on two non-randomised studies.

**Conclusions:**

The review findings are limited because studies include only people with Alzheimer’s-type dementia and lack confirmatory studies, although an increased risk of bleeding events is recognised. Further research that addresses the benefits and risks of aspirin in more representative groups of people with dementia is needed to guide prescribing decisions.

**Supplementary Information:**

The online version contains supplementary material available at 10.1007/s00228-021-03089-x.

## Introduction

More people than ever are living with dementia, and an increasingly recognised challenge is the management of comorbid disorders and risk states [1]. Risks and benefits of any medication may be different in people with dementia due to issues such as altered permeability at the blood-brain barrier, increased levels of frailty, and polypharmacy [2, 3], so recommendations for treatment or cessation of treatment (deprescribing) would ideally be made using evidence from people with dementia. The risk of major cardiovascular events (MACE), such as cerebrovascular accidents and myocardial infarctions, is related to many factors that are also risk factors for dementia, including age and cardiovascular disease [4]. The use of aspirin to prevent MACE is well-established, with a reduction of about 25% in MACE regardless of baseline risk, making it a valuable tool in preventing such events in people at a high risk of MACE [5, 6], but for people at a lower risk, the incidence of adverse events may negate any potential benefits [5, 7], with risks such as gastric irritation [8] and intracerebral haemorrhage (ICH) [9]. Studies specifically looking at older people at a low or medium risk of MACE have failed to show a consistent benefit of aspirin and have shown harm [10, 11], but these studies excluded participants with dementia.

Post-mortem examination of people who have died of dementia in old age typically finds both vascular and inflammatory lesions, regardless of clinical subtype allocated in life [12]. As aspirin is anti-inflammatory and anti-thrombotic, [6], it has been suggested that it may be useful in preventing or treating such lesions, but large trials and reviews of observational studies suggest that aspirin does not prevent dementia [13, 14] nor slow dementia labelled as Alzheimer’s disease type (AD) [15], while there is insufficient evidence on aspirin’s effect on progression of vascular dementia [6, 16]. There is no available evidence for other non-steroidal anti-inflammatory drugs or other anti-platelet drugs preventing or treating dementia [15, 17, 18]. Therefore, the main indication for people with dementia to take aspirin is to reduce MACE.

## Aims and objectives

This systematic review evaluates the evidence for the long-term effectiveness and harm of aspirin, compared to no anti-platelet, for people with dementia aged over 65 years. The primary outcome was reduction of MACE, but we also considered other possible benefits on general health and dementia progression, and possible harms. We considered evidence from observational studies as well as trials since recruitment and follow-up can be difficult in people with dementia due to frailty, high mortality, and difficulties maintaining consent [19]. We planned to investigate any demographic or clinical features that predict benefit and/or harm, including whether aspirin was for primary or secondary prevention, if suitable evidence was available.

## Methods

This review was conducted according to the Preferred Reporting Items for Systematic Reviews and Meta-Analyses (PRISMA) main statement and “harms” checklist [20, 21]. The checklist is available in Supplementary Table S1 (Online Resource). The review protocol is registered at **PROPSERO**: CRD42019144773, a copy of which is available as Supplemental Text (Online Resource) [22].

### Search strategy and selection criteria

Full details of search are in the protocol (Supplemental Text, Online Resource) and summarised here. We searched PubMed (Medline), Web of Science (Embase), and Cochrane trial databases using terms “dementia” and “aspirin or antiplatelet”, as described in the protocol with a time window of January 1, 2007, to November 9, 2020 (main search performed May 22, 2019, and “top-up” search performed November 9, 2020). The results were imported into endnote and de-duplicated. Forward and back citation searches of included papers and relevant systematic reviews were used. The search start date was chosen to maximise applicability to modern clinical practice, as the nature of dementia cohorts over time and between countries has changed, with a current emphasis on early diagnosis and use of anti-cholinesterase inhibiting medication [1].

Two investigators (KD, DB) independently screened abstracts (KD only for the “top-up” search) and discussed possible papers for inclusion with another author (RS) using the criteria in the protocol. We included longitudinal controlled studies (trials and observational), but due to the danger of confounding by indication in observational studies, we pre-specified that non-randomised studies must account for vascular risk in the design or analysis. The target population was people with dementia (all subtypes) where the mean age of the sample was over 65 years. Comorbid disease and other medication use were allowed. The exposure of interest was daily aspirin, whether prevalent or newly initiated (minimum 6 months). The comparator was no aspirin and no other anti-platelet pharmacological treatment, and allowed for studies of discontinuation (minimum 6 months). The outcomes had to be reported at a minimum of 2 years after the start of aspirin or the period of observation. Primary outcomes were:

i) Major cardiovascular events (MACE) or individual myocardial infarction, cerebrovascular accident, and transient ischaemic attack
ii) General health outcomes (mortality, hospital admission)

Secondary outcomes were:

iii) Dementia progression (clinical dementia rating or score on cognitive function scale)
iv) Secondary health outcomes (admission to care home, quality of life, falls, fractures, change in frailty, patient-reported outcomes)

Adverse events were counted at any time and included adverse drug events and drug interactions as counted by the study. Given concerns about bleeding events, including ICH, demonstrated in the literature, bleeding events were added post-protocol as a subject of particular interest.

### Data extraction and study quality

Data from included papers were extracted and risk of bias assessments carried out by KD in discussion with RS. For adverse events, it was pre-specified that all events reported would be extracted, as the designation of “treatment-related” events may not be consistent across studies. It was pre-specified that randomised controlled trials (RCTs) were assessed for risk of bias using the Cochrane Collaboration tool and all other studies by the Newcastle-Ottawa Quality Assessment Scale. The Cochrane Collaboration tool v2.0 [23] has five domains for risk of bias: (i) randomisation; (ii) deviation from intended interventions; (iii) missing outcome data; (iv) measurement of outcome; (v) selection of reported result. Each of the domains is rated “high risk”, “some concerns”, and “low risk”, with an overall rating of the same. The Newcastle-Ottawa Quality Assessment Scale for cohort studies [24] gives scores in the domains of (i) selection, (ii) comparability, and (iii) exposure/outcome and requires that we pre-define confounders that it would be preferable for studies to control for: we designated vascular risk as the primary, and age and other vascular medications as secondary confounders.

### Data synthesis

Results were tabulated and plotted, and effect sizes calculated (odds ratio for relative and events per 1000 for absolute) using Review Manager [25] and GRADE Pro [26], using the default Review Manager handling of zero event cells. We used 95% confidence intervals throughout. Clinical significance was set at an absolute increase or decrease of 10 or more per 1000.

The strength of the overall evidence for each finding was assessed with the Grading of Recommendations Assessment, Development and Evaluation (GRADE) tool [27] giving “high”, “moderate”, “low”, and “very low” confidence in findings. Evidence from RCTs starts at “high” and is then downgraded if found to have one of five indicators: (i) risk of bias (assessed described in “Data extraction and study quality”), (ii) inconsistency (between studies), (iii) indirectness (deviation from the population or intervention of the review), (iv) imprecision (confidence intervals cross clinical significance line), (v) other considerations (including publication bias). Evidence from non-randomised studies is first designated “low” quality, can be downgraded as per RCTs, but can also be upgraded in the presence of indicators that increase confidence: (i) large effect size; (ii) remaining confounding could only plausibly reduce effect size/reduce the chance of finding a significant result; (iii) dose sensitivity.

## Results

**Fig. 1:**
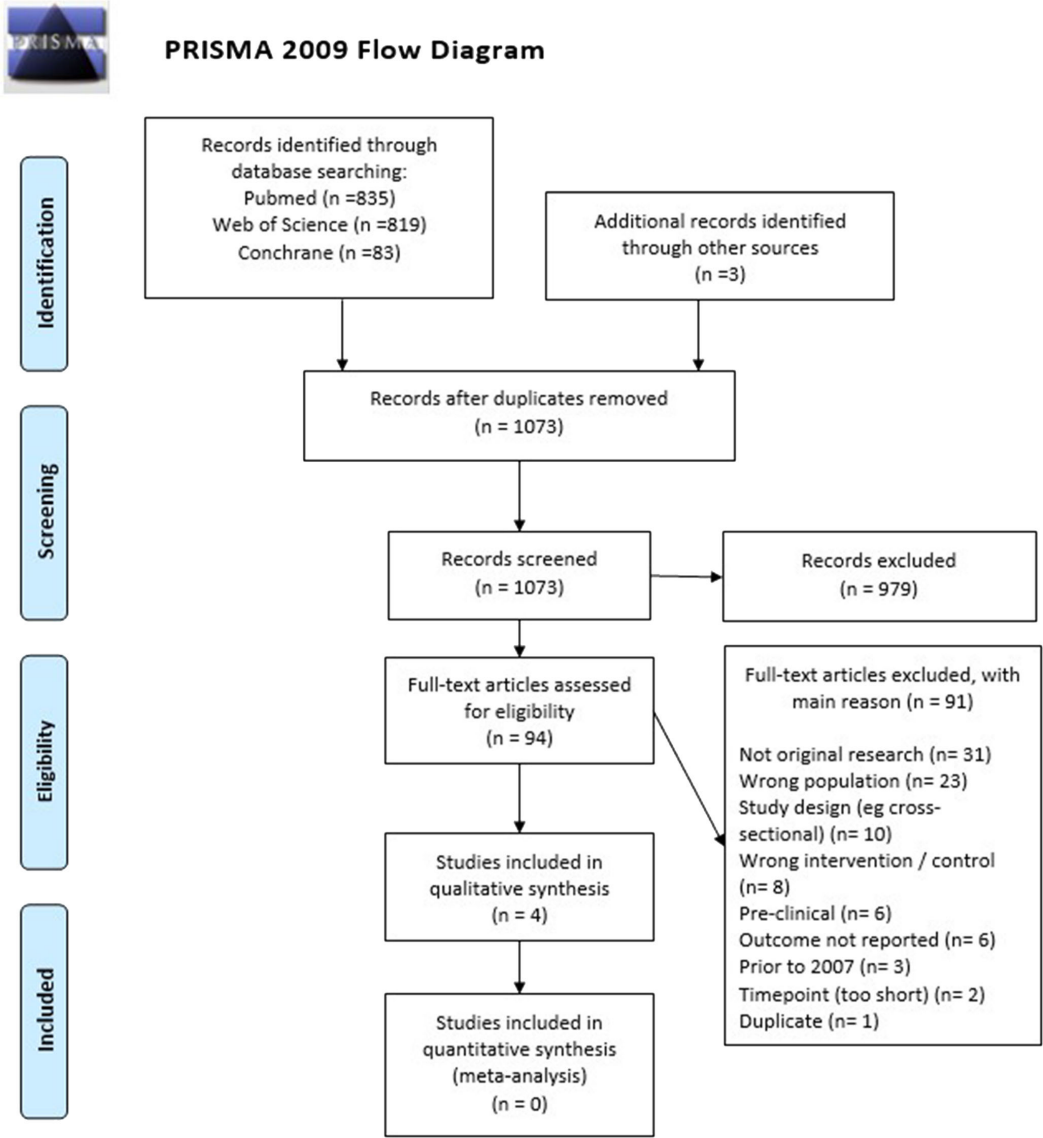
PRISMA flow diagram for inclusion in aspirin systematic review

From 1074 search results, 95 papers were selected for relevance, and four met eligibility criteria, as shown in Supplementary Table S2 (Online Resource) and Fig. 1. Table 1 describes these studies. An RCT named AD2000 from the UK [28] and a cohort study from Italy by Ferrari et al. [29] were designed to investigate dementia progression, and AD2000 also included general health outcomes and adverse events. The third and fourth studies only look at risk of ICH, using pooled data from two trials (including AD2000) [30] and a national health registry [31]. All studies included only people with AD subtype of dementia.

**Table 1:** Included studies

Main paper	A.D. Collaborative Group [28]	Thoonsen et al. [30]	Ferrari et al. [29]	Lee et al. [31]
Study type	AD2000 trial Open-label RCT 2 × 2 arms	Non-randomised data analysis from two trials (AD2000 and Richard)	Virtual cohort	Virtual cohort
Population	Multicentre trial recruited from memory clinics in the UK for a trial of donepezil and aspirin. Likely AD, with or without vascular component. Community-dwelling with a proxy informant. No indications for aspirin nor contraindications	331 people from the AD2000 trial and 123 from Richard et al.’s trial [32], a trial of enhanced vascular care for people with mild AD with vascular lesions on MRI	Patients with probable AD, or with possible AD with positive AD biomarkers, seen between 2009 and 2012 in a neurology clinic. Required at least a 2-year follow-up and genetic testing for ApoE	The Taiwan National Health Insurance Research Database, people with AD identified through diagnosis code or prescription, based on a random extract from the full database
Age	< 60 years, 5%; 60–69, 19%; 70–79, 51%; > 80 years, 25% Median 74 years	74 years AD2000, 76 years Richard	Mean 76 years 38% < 66 years	> 65 years, 93% in the anti-platelet group, 85% in the no anti-platelet group
Cardiovascular risk profile	Excluded those with indications for aspirin, which included hx of myocardial infarction, unstable angina, cerebrovascular accident, or transient ischaemic attack	AD2000: excluded those with indications for aspirin. Richard: not an exclusion. Required white matter lesion/s of vascular origin	All included. Vascular diseases were ascertained from medical documentation and testing done during assessment	Vascular risk factors extracted from claims history and used to create propensity score
Dementia subtype Dementia severity	Alzheimer’s dementia Mild-moderate	Alzheimer’s dementia Mild-moderate	Alzheimer’s dementia Any (average MMSE 22)	Alzheimer’s dementia Any (first recorded dementia event)
Intervention (*n*, %) Control (*n*, %)	Aspirin (156, 50%) Avoid aspirin (154, 50%)	Aspirin (156 + 65, 51%) Control (154 + 58, 49%)	Aspirin (73, 46%) No aspirin (87, 54%)	Anti-platelet (including aspirin 100 mg+) prescribed for > 3 months: 824 (with subgroup aspirin = 656) No more than one-off anti-platelet prescription after first dementia event: 824 matched
Length of intervention Background treatment	3 years (but sample size reduces after 1 year) Approximately half were also randomised to take donepezil	Mean time of follow-up: 29 months (AD2000), 22 months (Richard) Donepezil by around 50% (AD2000), 20% (Richard)	Prevalent use of aspirin (length unspecified) Any allowed. around 27% on statins, 44% on AChEI, and 29% on memantine	Prevalent or incident aspirin, for mean 22 months. Follow-up up to 12 years. Mean 4.8 years Any treatment except anti-coagulants allowed
Outcomes extracted (i–iv from methods)	Mortality: ascertained through follow-up and national records (24 months) Cognition: change in MMSE ascertained during follow-up assessments (36 months) Care-home entry: ascertained through follow-up (36 months) Adverse events: multiple side effects elicited by follow-up interviews and investigation of any hospitalisation or death (severe/severe bleeding)	Adverse: intracranial haemorrhage—ascertainment in AD2000 as for that cohort, unspecified for Richard	Cognition: “fast decline” classified through routine administration of MMSE at least 2 years apart. Fast decline if faster than median decline ≥ 4 pts. in 24 months (24 months)	Adverse: intracranial haemorrhage—ascertained through routine reporting in national insurance database

*AD*, dementia in Alzheimer’s disease; *MMSE*, mini-mental state exam out of 30; *RCT*, randomised controlled trial

AD2000 [28] was a multisite study that recruited 310 people with mild-moderate dementia without a vascular component from memory services in the UK. It was a 2 × 2 randomised study of donepezil and aspirin. Individuals with an indication for aspirin (cardio- or cerebro-vascular disease) were excluded from the aspirin aspect of the trial. The trial was open-label, with the treatment group advised to take 75-mg enteric-coated aspirin and the control group advised to avoid aspirin. It was not stated whether gastric protection was routinely used. At 2 years, 44% (33/75) of those allocated to aspirin arm had stopped and 11% (8/72) of those allocated to avoiding aspirin were taking it. In the risk of bias assessment, this “switching” between groups led to concerns in the deviation from intended treatment group category (Supplementary Table S3, Online Resource) with the intention-to-treat analysis, meaning that bias was likely to be in the direction of not finding a true effect; a high loss to follow-up was also noted. Thoonsen et al. [30] conducted an individual patient data meta-analysis of 2 trials: AD2000 and Richard et al.’s trial of enhanced vascular care in 130 people with AD and vascular lesions, where aspirin was not randomised [32].

A study by Ferrari et al. [29] was a virtual cohort study from electronic records of a neurology service in Florence consisting of 160 people with AD who had routine mini-mental state assessments (MMSE) recorded 2 years apart. The study categorised people into fast or slow declining MMSE based on the median MMSE decline of the cohort. Aspirin use was one of a number of predictor variables. The dose of aspirin and use of gastric protection were not mentioned. In the risk of bias assessment, the study lost three points for lack of clarity over as follows: (i) how the use of aspirin was ascertained; (ii) whether assessors were blinded to aspirin usage; and (iii) how the analysed sample differed from those who were not followed for 2 years (Supplementary Table 4, Online Resource). Ferrari et al. examined prevalent aspirin use, whereas AD2000 examined incident aspirin use. Those already taking aspirin in Ferrari et al. would likely have been excluded from AD2000, as they likely had indications for aspirin (an exclusion criterion in AD2000). Lee et al. [31] reported an observational study using the Taiwanese national population database, examining whether anti-platelets in dementia increase the risk of ICH by comparing those taking aspirin after a dementia diagnosis to matched samples of people without dementia or not taking anti-platelets. On risk of bias assessment, the paper lost only one point, for not adjusting for other medications (Supplementary Table 4, Online Resource).

### Findings

#### Major cardiovascular events

MACE were not reported by any papers, except for ICH, which is considered in the adverse events section.

#### General health outcomes and secondary health outcomes

The AD2000 trial investigated mortality (primary outcome in this review) and admission to care home (secondary outcome), as shown in Table 2 and Supplementary Figures S1 and S2 (Online Resource). During the first 2 years of the study, 17% of the cohort died, with deaths being approximately equal in the two arms (odds ratio 1.07 (95% CI 0.58–1.97) for aspirin vs. control). Over the course of the trial, 52% of the surviving participants entered a care home, with no significant difference between the two groups (*hazard* ratio 0.94 (95% CI 0.67–1.31) for aspirin vs. control). The findings of no effect on general health were rated “very low” confidence in Table 2.

**Table 2:** GRADE table for health outcomes, aspirin vs. control

Certainty assessment							No. of patients		Effect of aspirin vs. control			Confidence in efficacy statement
No. of studies	Study design	Risk of bias	Inconsistency	Indirectness	Imprecision	Other	Aspirin	Avoid aspirin	Relative (95% CI)	Absolute (95% CI)	Efficacy statement	
Deaths (follow-up: mean 24 months)												
1	Randomised trials	Serious^a^	Not serious	Serious^b^	Very serious^c^	None	26/150 (17.3% mortality)	24/147 (16.3% mortality)	OR 1.07 (0.58 to 1.97)	9 more deaths per 1000 (from 62 fewer to 114 more)	Aspirin has no significant effect on deaths	⨁◯◯◯ Very low
Entry into care home (follow up: mean 36 months)												
1	Randomised trials	Serious^a^	Not serious	Serious^b^	Very serious^c^	None	156	154	HR 0.94 (0.67 to 1.31)	22 fewer entry per 1000 (from 132 fewer to 98 more)	Aspirin has no significant effect on entry into care home	⨁◯◯◯ Very low
								52.0% care home entry				

*CI*, confidence interval; *OR*, odds ratio; *HR*, hazard Ratio; *MD*, mean difference

^a^Cochrane risk of bias tool highlighted risk from deviation from intended treatment (includes lack of blinding) and missingness

^b^Includes only those with Alzheimer’s disease and without high vascular risk

^c^Confidence interval crosses lines of clinical importance on both benefit and harm

#### Dementia progression

Table 3 shows two results regarding dementia progression and aspirin, both of which used MMSE to monitor dementia progression over a period of 24 months. The control arm of AD2000 had a mean deterioration of 5.0 pt., while the aspirin arm had a mean deterioration of 4.8 pt. The mean difference was not significantly different (0.2 pt., 95% CI from 2.4 pts. worse to 2.6 pts. better in aspirin vs. control) as shown in Supplementary Figure S3 (Online Resource). In Ferrari et al., the fully adjusted model found that the odds of someone with AD having a rapid decline of MMSE were lower in the group taking aspirin (OR 0.34, 95% CI 0.11–0.88). In Table 3, the GRADE ratio for the finding of no effect from AD2000 was “very low”, and the finding of beneficial effect from the Ferrari cohort was also “very low”.

**Table 3:** GRADE tables for dementia progression, aspirin vs. control

Certainty assessment							No. of patients		Effect of aspirin vs control			Confidence in efficacy statement
No. of studies	Study design	Risk of bias	Inconsistency	Indirectness	Imprecision	Other considerations	Aspirin	No aspirin	Relative (95% CI)	Absolute (95% CI)	Efficacy statement	
MMSE decline (follow up: mean 24 months)												
1	Randomised trials	Serious^a^	Not serious	Serious^b^	Serious^c^	Inconsistency with below	83	86	-	MD 0.2 pts. less decline (2.4 more to 2.6 less)	Aspirin has no significant effect on MMSE decline	⨁◯◯◯ Very low
							4.8 pt. decline	5.0 pt. decline				
Rapid MMSE decline (follow-up: mean 24 months; assessed with above median decline of 2 pts. in first year and 4 pts. in 2 years)												
1	Observational studies	Serious^d^	Not serious	Serious^e^	Not serious	Strong association; inconsistency with above	73	87	OR 0.34 (0.11 to 0.88)	262 fewer rapid decliners per 1000 (from 31 fewer to 458 fewer)	Aspirin protects against rapid MMSE decline	⨁◯◯◯ Very low
							Adjusted model	Adjusted model				

*CI*, confidence interval; *MD*, mean difference; *OR*, odds ratio

^a^Cochrane risk of bias tool highlighted risk from deviation from intended treatment (includes lack of blinding) and missingness

^b^Includes only those with Alzheimer’s disease and without high vascular risk

^c^Although this measure of difference does not have pre-specified line of clinical effect, the authors considered that > 2.5-pt. difference on MMSE was significant, and therefore, this was imprecise

^d^Newcastle-Ottawa assessment scale highlighted concern re-ascertainment of exposure, no detail on those followed up vs. not followed up and no mention of assessor blinding

^e^Includes only those with Alzheimer’s disease who were followed up

#### Adverse events

The AD2000 trial reported all adverse events in both arms as shown in Table 4 and Supplementary Figure 4 (Online Resource). Significantly more people in the aspirin arm experienced any adverse event (OR 1.89, 1.20–2.97), but not serious adverse events (OR 1.33, 0.84–2.11) or mortality (see “General health outcomes and secondary health outcomes”). Severe bleeding events (requiring hospital admission or fatal) were significantly more common in the aspirin arm (OR 6.91, 1.53–31.15), including four gastric bleeds in the aspirin arm and one in the control arm. Thoonsen’s pooled analysis shows seven (7/221) ICH in people taking aspirin and none (0/212) in the control group, the equivalent of 30 extra cases per 1000. This gives a large odds ratio with very wide 95% confidence intervals (OR 14.86, 0.83 to 250.43). The Lee registry study found that having dementia was significantly associated with ICH. Compared with matched controls who had no dementia diagnosis and were not prescribed anti-platelets, individuals with AD who were not prescribed anti-platelets have a hazard ratio for ICH of 2.02 (1.10–3.72) and individuals with AD who were prescribed aspirin have a hazard ratio for ICH of 2.22 (1.07–4.62). Comparing these two groups suggests an extra risk of ICH of around one case per thousand people with dementia taking aspirin (from 15 less to 20 more). The GRADE ratings for aspirin causing any adverse events and severe bleeding events were “moderate” (Table 4), but the GRADE ratings for aspirin not causing a clinically significant increase in serious adverse events and ICH compared to placebo were “very low” since the risk of bias assessment had shown that the risk was in the direction of preventing the detection of a real difference between the groups.

**Table 4:** GRADE table for adverse events, aspirin vs. control

Certainty assessment							No. of events/patients (% experienced)		Effect of aspirin vs. control			Confidence in efficacy statement
No. of studies	Study design	Risk of bias	Inconsistency	Indirectness	Imprecision	Other	Aspirin	No aspirin	Relative (95% CI)	Absolute (95% CI)	Efficacy statement	
Adverse events—any												
1	RCT	Not serious^a^	Not serious	Serious^b^	Not serious	None	82/156 (52.6%)	57/154 (37.0%)	OR 1.89 (1.20 to 2.97)	156 more per 1000 (from 43 more to 266 more)	Aspirin increases risk of adverse events	⨁⨁⨁◯ Moderate
Adverse events—serious												
1	RCT	Serious^a^	Not serious	Serious^b^	Very serious^c^	None	63/156 (40.4%)	52/154 (33.8%)	OR 1.33 (0.84 to 2.11)	66 more per 1000 (from 38 fewer to 181 more)	Aspirin has no significant effect on the risk of serious adverse events	⨁◯◯◯ Very low
Adverse events—severe bleeding												
1	RCT	Not serious^a^	not serious	Serious^b^	Not serious	None	13/156 (8.3%)	2/154 (1.3%)	OR 6.91 (1.53 to 31.15)	70 more per 1000 (from 7 more to 278 more)	Aspirin increases risk for severe bleeding	⨁⨁⨁◯ Moderate
Adverse events—intracranial haemorrhage												
3^d^	Non-RCT	Serious^d^	Not serious	Serious^e^	Not applicable^f^	None	Pooled data from trials: aspirin group 7/221 (3.2%), control group 0/212 (0%) OR 14.86 (0.83 to 250.43) 30 more per 1000 (0.2 less to 353 more)^g^ Registry data (after adjustments and competing risk correction): compared to no dementia/no aspirin—aspirin group HR 2.22 (1.07–4.62), control group HR 2.02 (1.10–3.72) Est. numbers of ICH—aspirin 9 (4–19)/3476 person-years, control group 11 (6–20)/4452 person-years Est. absolute difference—1 more per 1000 people (15 less to 20 more)				Aspirin has no statistically significant effect on the risk of intracranial haemorrhage	⨁◯◯◯ Very low

*CI*, confidence interval; *OR*, odds ratio

^a^Cochrane risk of bias tool highlighted high levels of deviation from intended treatment (decreases confidence in null findings but not positive findings as intention-to-treat analysis used)

^b^Includes only those with Alzheimer’s disease and without high vascular risk

^c^Confidence interval crosses lines of clinical importance on both benefit and harm

^d^Includes pooled randomised and non-randomised data (Thoonsen et al.), primarily randomised from AD2000 (risk of bias felt possible from deviation from intended treatment) and registry study (Lee et al., observational but low formal risk of bias)

^e^Includes only those with Alzheimer’s disease

^f^Studies are not combined; however, it is noted that estimates from one study cross the line of clinically significant harm, and the other study crosses the lines of both significant harm and significant benefit

^g^Effect rare and calculation of absolute effect confidence intervals are based on estimates of background rate based on 1:1000 over 2 years

^f^As calculated by Cochrane Review Manager

## Discussion

This review of studies investigating the long-term efficacy and safety of aspirin in people with dementia found four reports, all concentrating on AD dementia. For the primary outcome of MACE, there was no evidence identified. Other efficacy outcomes had evidence, but the confidence in the findings was rated “very low”. A protective effect on dementia progression was seen in a cohort study (Ferrari et al. [29]) of prevalent aspirin use, but not in a trial of aspirin initiation. (AD2000 [28]). The trial also found no significant differences in mortality but an excess of serious bleeding events in the aspirin group.

No clear conclusion can be drawn on the efficacy of aspirin. The difference between the trial findings [28] and the cohort study [29] on dementia progression could be an artefact due to different measures or chance. However, an important difference is that the trial excluded anyone with an indication for aspirin, stating that “almost 50% of patients were ineligible, most commonly because of a potential indication for aspirin [secondary prophylaxis after myocardial infarction, unstable angina, or a cerebral transient ischaemic attack]”, which means that although the control group of the trial and cohort studies might be equivalent, the group taking aspirin was different, as the people from the cohort study who happen to be on aspirin at time of dementia diagnosis can be assumed to have an indication for aspirin, most likely vascular disease. Although the cohort study corrects for vascular disease and cerebrovascular pathology in the analysis, this may not have been sufficient to correct confounding by indication. Second, those who are on aspirin at diagnosis can be assumed to have been taking it beforehand, whereas those in the trial were newly started on aspirin. Aspirin at a pre-clinical stage of dementia and a greater vascular component to the overall clinical condition could plausibly contribute to greater efficacy of aspirin. A possible inference may be that aspirin is unlikely to help when used as primary prevention (as per the trial) but may affect the rate of decline in dementia where it is otherwise indicated (as per the cohort study). This interpretation concurs with recent studies suggesting that aspirin did not appear to reduce the incidence of dementia in people who did not have any other indication for aspirin (Aspirin in Reducing Events in the Elderly Trial, ASPREE) [13].

The studies in this review involved only people with AD. This may be because aspirin treatment is a standard care for those with vascular dementia. However, two studies in vascular dementia from the literature that were not included as they fell outside our search window show conflicting results regarding the potential benefit: an RCT (Meyer et al. 1989) [33] found aspirin to be beneficial on cognitive outcomes, but an observational study (Devine and Rands 2003) [34] found no benefit on time-to-death-or-institutionalisation.

For adverse events, mostly we relied on the report of the AD2000 trial [28]. There was evidence that people in the aspirin arm had more adverse events, but not significantly more serious events or deaths. The finding that they had significantly more severe bleeding events is credible because it agrees with other studies of aspirin in older people [11]. A weakness of these studies is the lack of information given about concomitant medication such as proton pump inhibitors, selective serotonin uptake inhibitors, and steroids that may have increased or decreased risk of bleeding [35, 36]. It seems likely that there is a higher risk of intracerebral haemorrhage despite the lack of statistical significance in both studies [9, 30].

This review finds that there is no evidence that initiating aspirin in someone with dementia with no vascular risk factors will have benefit, but may cause harm. This accords with the 2019 Beers Criteria of potentially inappropriate medication in older people, which advises “extra caution” in prescribing aspirin to people aged 70 or above for primary prevention [37]. However, there are a number of uncertainties, for instance, what to do if someone already has aspirin prescribed or if their dementia has cerebrovascular pathology. Clinical uncertainty also arises because people with dementia present often with complex multimorbidity and polypharmacy [2]. Adopting medication reviews in people with recently diagnosed dementia would allow for the indications for aspirin to be recorded, letting clinicians evaluate individual risk/benefit profiles and utilise available evidence-based guidance, taking into account patient preferences [38–40]. It may not be ethical or practical to randomise people with dementia with indications for aspirin to a placebo-controlled trial, but observational studies may be able to leverage variation in treatment as a “natural experiment” to obtain real-world data [20] and close this knowledge gap.

### Strengths and limitations

To our knowledge, this is the first review of the use of aspirin in dementia that has included observational data, potentially adding real-world evidence. We have tried to address possible weaknesses in observational studies by requiring longitudinal data with a control arm and correction for the main indicator/confounder, vascular risk.

A limitation is that there were few studies to review, partially due to search criteria that aimed to maximise the applicability of any findings to the current clinical dilemma and restricted to published data. The studies that we found varied in quality and all had at least one potential item of concern on risk of bias assessment. We were only able to study AD and were unable to find any data on MACE. We were unable to further examine data for characteristics that may predict benefit and harm. Lastly, we looked only at aspirin, whereas other anti-platelet medications may have clinically relevant differences.

## Conclusions

There are inadequate data available to make an informed recommendation regarding the prescribing or de-prescription of aspirin in dementia, except to say it is likely that there is an increased risk of clinically important bleeding events in individuals with AD receiving aspirin. Given the established efficacy for preventing MACE in people with high vascular risk, clinicians are likely to continue considering aspirin for those with dementia and comorbidities. The outcomes of these decisions, captured in electronic health records, should be used for creating more applicable evidence through well-designed observational studies to help ensure that people with dementia get the most appropriate treatment.

## Supplementary information

ESM 1 (DOCX 159 kb)

## Data Availability

All data generated or analysed during this study are included in this published article (and its Online Resource supplementary tables).

## References

[1] Alzheimer’s Disease International, World Health Organization (2012) Dementia: a public health priority. Department of Mental Health and Substance Abuse. http://www.who.int/mental_health/publications/dementia_report_2012. Accessed 21 July 2020

[2] Bishara D, Harwood D (2014). Safe prescribing of physical health medication in patients with dementia. Int J Geriatr Psychiatry.

[3] Eshetie TC, Nguyen TA, Gillam MH, Kalisch Ellett LM (2018). A narrative review of problems with medicines use in people with dementia. Expert Opin Drug Saf.

[4] Hippisley-Cox J, Coupland C, Brindle P (2017). Development and validation of QRISK3 risk prediction algorithms to estimate future risk of cardiovascular disease: prospective cohort study. BMJ..

[5] Antiplatelet Trialists’ Collaboration (1994). Collaborative overview of randomised trials of antiplatelet therapy--I: Prevention of death, myocardial infarction, and stroke by prolonged antiplatelet therapy in various categories of patients. Antiplatelet Trialists’ Collaboration. BMJ (Clin Res Ed).

[6] Hybiak J, Broniarek I, Kiryczynski G, Los LD, Rosik J, Machaj F (2020). Aspirin and its pleiotropic application. Eur J Pharmacol.

[7] Baigent C, Blackwell L, Collins R, Emberson J, Godwin J, Antithrombotic Trialists Collaboration (2009). Aspirin in the primary and secondary prevention of vascular disease: collaborative meta-analysis of individual participant data from randomised trials. Lancet..

[8] Jorgensen PW, Calleja EL, Gaso PS, Matarranz del Amo M, Navarro RA, Sanchez JM (2011). Antiagregation and anticoagulation, relationship with upper gastrointestinal bleeding. Rev Esp Enferm Dig.

[9] Gorelick PB, Weisman SM (2005). Risk of hemorrhagic stroke with aspirin use: an update. Stroke.

[10] Sugawara M, Goto Y, Yamazaki T, Teramoto T, Oikawa S, Shimada K (2019). Low-dose aspirin for primary prevention of cardiovascular events in elderly Japanese patients with atherosclerotic risk factors: subanalysis of a randomized clinical trial (JPPP-70). Am J Cardiovasc Drugs.

[11] McNeil JJ, Wolfe R, Woods RL, Tonkin AM, Donnan GA, Nelson MR, Reid CM, Lockery JE, Kirpach B, Storey E, Shah RC, Williamson JD, Margolis KL, Ernst ME, Abhayaratna WP, Stocks N, Fitzgerald SM, Orchard SG, Trevaks RE, Beilin LJ, Johnston CI, Ryan J, Radziszewska B, Jelinek M, Malik M, Eaton CB, Brauer D, Cloud G, Wood EM, Mahady SE, Satterfield S, Grimm R, Murray AM, ASPREE Investigator Group (2018). Effect of aspirin on cardiovascular events and bleeding in the healthy elderly. N Engl J Med.

[12] Montine TJ, Sonnen JA, Montine KS, Crane PK, Larson EB (2012). Adult Changes in Thought study: dementia is an individually varying convergent syndrome with prevalent clinically silent diseases that may be modified by some commonly used therapeutics. Curr Alzheimer Res.

[13] Ryan J, Storey E, Murray AM, Woods RL, Wolfe R, Reid CM, Nelson MR, Chong TTJ, Williamson JD, Ward SA, Lockery JE, Orchard SG, Trevaks R, Kirpach B, Newman AB, Ernst ME, McNeil JJ, Shah RC, on behalf of the ASPREE Investigator Group (2020). Randomized placebo-controlled trial of the effects of aspirin on dementia and cognitive decline. Neurology..

[14] Veronese N, Stubbs B, Maggi S, Thompson T, Schofield P, Muller C, Tseng PT, Lin PY, Carvalho AF, Solmi M (2017). Low-dose aspirin use and cognitive function in older age: a systematic review and meta-analysis. J Am Geriatr Soc.

[15] Jaturapatporn D, Isaac MGEKN, McCleery J, Tabet N (2012) Aspirin, steroidal and non‐steroidal anti‐inflammatory drugs for the treatment of Alzheimer's disease SO. Cochrane Database Syst Rev 1465–1858. 10.1002/14651858.CD006378.pub2 10.1002/14651858.CD006378.pub2 PMC11337172 22336816

[16] Rands G, Orrel M, Spector A, Williams P (2004) Aspirin for vascular dementia SO. Cochrane Database Syst Rev 1465–1858. 10.1002/14651858.CD00129610.1002/14651858.CD00129610796639

[17] McHutchison C, Blair GW, Appleton JP, Chappell FM, Doubal F, Bath PM, Wardlaw JM (2020). Cilostazol for secondary prevention of stroke and cognitive decline systematic review and meta-analysis. Stroke..

[18] Jordan F, Quinn TJ, McGuinness B, Passmore P, Kelly JP, Smith CT et al (2020) Aspirin and other non-steroidal anti-inflammatory drugs for the prevention of dementia. Cochrane Database Syst Rev 4. 10.1002/14651858.CD011459.pub2 10.1002/14651858.CD011459.pub2 PMC7192366 32352165

[19] Davis KA, Farooq S, Hayes JF, John A, Lee W, MacCabe JH et al (2019) Pharmacoepidemiology research: delivering evidence about drug safety and effectiveness in mental health. Lancet Psychiatry. 10.1016/S2215-0366(19)30298-6 10.1016/S2215-0366(19)30298-6 31780306

[20] Moher D, Liberati A, Tetzlaff J, Altman DG, The PG (2009). Preferred reporting items for systematic reviews and meta-analyses: the PRISMA statement. PLoS Med.

[21] Zorzela L, Loke YK, Ioannidis JP, Golder S, Santaguida P, Altman DG, Moher D, Vohra S, PRISMA harms group (2016). PRISMA harms checklist: improving harms reporting in systematic reviews. BMJ..

[22] Davis KAS, Bishara D, Molokhia M, Perera G, Stewart R (2019) Aspirin in people with dementia, long-term benefits and harms: protocol for a systematic review. In: International prospective register of systematic reviews. National Institute for Health Research. https://www.crd.york.ac.uk/prospero/display_record.php?ID=CRD42019144773. Accessed 21 July 2020

[23] Sterne J, Savović J, Page M, Elbers R, Blencowe N, Boutron I (2019). RoB 2: a revised tool for assessing risk of bias in randomised trials. BMJ.

[24] Wells G, Shea B, O’Connell D, Peterson J, Welch V, Losos M (2019). The Newcastle-Ottawa Scale (NOS) for assessing the quality of nonrandomised studies in meta-analyses. Clinical Epidemiology.

[25] The Nordic Cochrane Centre (2014) Review Manager (RevMan) [Computer program]. In: The Cochrane Collaboration, editor. Copenhagen

[26] Evidence Prime Inc (2015) GRADEpro GDT: GRADEpro Guideline Development Tool [Software]. In: McMaster University, editor. Available from gradepro.org

[27] Higgins J, Green S (eds) (2011) Cochrane handbook for systematic reviews of interventions version 5.1.0 (updated March 2011). The cochrane collaboration. Available from https://handbook.cochrane.org. Archived version https://training.cochrane.org/handbook/archive/v5.1/. Accessed 21 Jan 2021

[28] A. D. Collaborative Group (2008). Aspirin in Alzheimer’s disease (AD2000): a randomised open-label trial. Lancet Neurol.

[29] Ferrari C, Lombardi G, Polito C, Lucidi G, Bagnoli S, Piaceri I, Nacmias B, Berti V, Rizzuto D, Fratiglioni L, Sorbi S (2018). Alzheimer’s disease progression: factors influencing cognitive decline. J Alzheimers Dis.

[30] Thoonsen H, Richard E, Bentham P, Gray R, van Geloven N, De Haan RJ (2010). Aspirin in Alzheimer’s disease: increased risk of intracerebral hemorrhage: cause for concern?. Stroke..

[31] Lee TL, Liu CH, Chang YM, Lin TY, Chien CY, Chen CH, Tsai KJ, Lin SH, Sung PS (2020). The impact of antiplatelet use on the risk of intracerebral hemorrhage in patients with Alzheimer’s disease: a nationwide cohort study. J Alzheimers Dis.

[32] Richard E, Kuiper R, Dijkgraaf MG, Van Gool WA (2009). Vascular care in patients with Alzheimer’s disease with cerebrovascular lesions-a randomized clinical trial. J Am Geriatr Soc.

[33] Meyer JS, Rogers RL, McClintic K, Mortel KF, Lotfi J (1989). Randomized clinical trial of daily aspirin therapy in multi-infarct dementia: a pilot study. J Am Geriatr Soc.

[34] Devine M, Rands G (2003). Does aspirin affect outcome in vascular dementia? A retrospective case-notes analysis. Int J Geriatr Psychiatry.

[35] Lanas A, Gargallo CJ (2015). Management of low-dose aspirin and clopidogrel in clinical practice: a gastrointestinal perspective. J Gastroenterol.

[36] Moayyedi P, Eikelboom JW, Bosch J, Connolly SJ, Dyal L, Shestakovska O (2019). Safety of proton pump inhibitors based on a large, multi-year, randomized trial of patients receiving rivaroxaban or aspirin. Gastroenterology.

[37] By the American Geriatrics Society Beers Criteria® Update Expert Panel (2019). American Geriatrics Society 2019 Updated AGS Beers Criteria® for potentially inappropriate medication use in older adults. J Am Geriatr Soc.

[38] Bishara D, Scott C, Stewart R, Taylor D, Harwood D, Codling D, Banwell C, Sauer J (2020). Safe prescribing in cognitively vulnerable patients: the use of the anticholinergic effect on cognition (AEC) tool in older adult mental health services. BJPsych Bulletin.

[39] McGrattan M, Ryan C, Barry HE, Hughes CM (2017). Interventions to improve medicines management for people with dementia: a systematic review. Drugs Aging.

[40] Reeve E, Simon Bell J, Hilmer SN (2015). Barriers to optimising prescribing and deprescribing in older adults with dementia: a narrative review. Curr Clin Pharmacol.

