# Supplementary material for: Aspirin in people with dementia, long-term benefits, and harms: a systematic review
Source: Eur J Clin Pharmacol. 2021 Jan 22;77(7):943–54. doi: 10.1007/s00228-021-03089-x (PMC8184554; doi:10.1007/s00228-021-03089-x)
Supplement: Supplementary file 1 — (DOCX 159 kb) [file 228_2021_3089_MOESM1_ESM.docx]

Supplemental Material for paper:

**Aspirin in people with dementia, long-term benefits and harms: A systematic review**

**European Journal of Clinical Pharmacology**

**Katrina A. S. Davis, Delia Bishara, Mariam Molokhia, Christoph Mueller, Gayan Perera and**

**Robert J. Stewart**

**Corresponding author:**

Katrina Davis

Affiliations: *King's College London Institute of Psychiatry, Psychology and Neuroscience & South London and Maudsley NHS Foundation Trust*

Email: [katrina.davis@kcl.ac.uk](mailto:katrina.davis@kcl.ac.uk)

Supplemental text 1: Prospero review protocol

[Supplementary Figure S1 Aspirin vs control: Deaths at 2y 7](#_Toc51839889)

[Supplementary Figure S2 Aspirin vs control: Admission to care home at 3y 7](#_Toc51839890)

[Supplementary Figure S3 Aspirin vs control: MMSE decline at 2y 7](#_Toc51839891)

[Supplementary Figure S4 Aspirin vs control: Adverse events during trial 8](#_Toc51839892)

[Supplementary Table S1 (a) PRISMA harms checklist (b) Numbers for searches disaggregated 9](#_Toc59437379)

[Supplementary Table S2 All studies considered at "full-text" level, and decision about inclusion status. 17](#_Toc59437380)

[Supplementary Table S3 Risk of bias assessment for AD2000 21](#_Toc59437381)

[Supplementary Table S4 Risk of bias assessment for observational studies (Ferrari 2018 and Lee 2020) 22](#_Toc59437382)

Supplemental File 1: Prospero review protocol

**Aspirin in people with dementia, long-term benefits and harms: protocol for a systematic review**

**Introduction**

Many people with dementia also have vascular risk factors making them eligible for treatment with aspirin for primary prevention of coronary artery disease or secondary prevention of cerebrovascular accidents and myocardial infarctions. Due to the protective effect of aspirin on the vasculature and the vascular contribution to many cases of dementia, it has been thought that aspirin might reduce the risk of dementia or slow cognitive decline in established cases. However the best synthesis of current evidence, a Cochrane report updated in 2012, found evidence suggesting that aspirin did not improve the symptoms or slow progression in Alzheimer's dementia, and no evidence regarding possible benefit of aspirin on symptoms or progression of vascular dementia (Rands, Orrel et al. 2004, Jaturapatporn, Isaac et al. 2012). Aspirin is not without its risks; it often causes gastric discomfort, and there is a risk of gastric bleeding, such that a second agent (usually a proton pump inhibitor) is often prescribed in combination to reduce the gastric adverse effects of aspirin.

Guidelines on reducing cardiovascular risk often lead to older people being prescribed aspirin and other preventative medication indefinitely, which contributes to polypharmacy, especially if a proton pump inhibitor is needed. People with dementia in the UK take an average 5.4 medications (Clague, Mercer et al. 2016). Polypharmacy can be costly, confusing and dangerous. Higher number of prescribed medication in people with dementia has been found to be associated with more hospitalisations and earlier death (Mueller, Molokhia et al. 2018). The differing handling of medication in people with dementia means that their overall benefit may be reduced and risks may be increased, compared to other older people (Bishara and Harwood 2014, Eshetie, Nguyen et al. 2018). Given this, it may be that the added burden of taking aspirin exceeds the benefit derived once a life-limiting diagnosis such as dementia has been made.

Preliminary searches in PROSPERO and Cochrane systematic review database showed four systematic reviews related to dementia and aspirin. The two Cochrane reviews discussed above were found. In addition, a further review of anti-inflammatory agents, including aspirin, to treat Alzheimer's disease dementia conducted in 2018 was found, but this has not yet been published (Zhang and Wu 2018). All three of these studies reviewed randomised controlled trials (RCTs) only. A systematic review of observational trials looking at NSAIDs, aspirin and paracetamol (acetaminophen) for the prevention of Alzheimer's disease published in 2018 was also retrieved (Zhang, Wang et al. 2018). This study found 16 cohort studies including 236,022 participants published between 1995 and 2016, and found a relative risk of Alzheimer's dementia of 0.81 when taking any medication, but with fairly wide confidence intervals, such that for individual agents the null hypothesis fell within the 95% confidence interval – for aspirin RR 0.89, 95% CI 0.70 to 1.13.

This review is different in scope to the four found. First, it will be looking at people with dementia, unlike the review of observational studies above where people were free of dementia at baseline. Secondly it will be looking at the use of aspirin for its primary purpose of reducing risk of cardiac events and strokes rather than its effect on cognition / dementia progression. Thirdly it will look at both randomised controlled studies and observational studies. Randomised controlled trials can be difficult in this group, due to increased frailty and high mortality, putting a high burden on participants and making it difficult to retain participants, and subsequent high costs. There can also be problems with participants' capacity to consent to research as cognition declines. Therefore, it is valid to explore whether observational research can add to the evidence base.

**Objective**

The aim of the systematic review is to evaluate the evidence for the long term effectiveness and harm of aspirin therapy prescribed for cardiovascular health specifically for people with dementia aged 65 years or older compared to no antiplatelet treatment. If evidence allows, it will also look at whether there are clinical or demographic features that predict benefit and/or harms.

**Methods**

**Eligibility criteria**

- Study designs

We will include randomised controlled trials, including cluster RCTs, controlled clinical trials or cluster trials of both starting or discontinuation studies subject to duration requirements. Retrospective observational trials of comparative cohort studies and case-control or nested case-control studies will be included. Cross-sectional studies will be excluded, along with cross-over studies, interrupted time series and controlled before-after studies due to the emphasis of long-term effects.

- Participants

Participants of studies must include a majority of people with a diagnosis of dementia (or any dementia subtype) or a majority of people with a diagnosis of MCI and at least 10% of people with dementia. Younger onset dementia is likely to vary significantly in terms of risks, therefore studies must also have 80% of participants over 64 or described as “old” or “elderly”. If a larger study reports a subgroup of people with dementia, this will only be considered if pre-specified, and age requirement are met. Comorbidity would not be an exclusion criterion.

- Interventions

Intervention will include aspirin, or aspirin plus another anti-platelet, or aspirin with other medications to reduce vascular risk (e.g. polypill with statin) at a conventional dose (75 - 300mg daily). Aim of aspirin must be for primary or secondary prevention, not for atrial fibrillation or pain. RCTs of discontinuation and observational studies of both incident use and prevalent use would be suitable, as long as aspirin is continued or stopped for at least six months. Concomitant medication would not be an exclusion criterion but use of combined medication will be considered separately in sensitivity analyses.

- Comparison

The control group will be no aspirin. Since the aim is not to compare different antiplatelets, only a control group involving another antiplatelet would be inadequate.

- Outcomes

Outcomes at a minimum of two years after start of aspirin or period of observation, except for adverse drug events and drug interactions, which can be at any time-point. Outcomes must include at least one of:

• heart attack / myocardial infarction

• stroke / CVA

• transient ischaemic attack

• unplanned hospital admissions

• death

• dementia progression (clinical dementia rating, MMSE, or other stated score/rating)

• admission to care home

• adverse drug event (as defined by study)

• drug interaction (as defined by study).

Composite measures of the above may also be collected (see Study Records).

- Setting

Studies will be restricted to papers published in the last twelve years and taking place in developed nations, since the care of vascular risk factors and the circumstances of people with dementia are likely to differ substantially outside of this.

- Language

The study must be published in some form in English.

- Publication

Preference will be given to studies that have published in peer-review journals. However, if there are less than five studies in any of the primary outcomes, then literature returned in the searches below that is not peer-reviewed will be screened for inclusion.

**Search**

We will search PubMed (Medline), Web of Science (Embase) and Cochrane trial database with a date limit of twelve years and a language limit of English. No study design limits will be imposed. If we find reviews, we will carry out forward and back citation searches to strengthen recall of relevant papers. Due to resource limitations, we do not plan to contact study authors or search grey literature. An example search for medline is reproduced below.

Search **(((Dementia[MeSH Terms] OR Cognitive dysfunction[MeSH Terms] OR Dementia[Title/Abstract] OR Alzheimer*[Title/Abstract]))) AND ((aspirin[Title/Abstract]) OR "Platelet Aggregation Inhibitors" [Pharmacological Action])** Filters: **published in the last 12 years**

**Study records**

Sifting and screening for eligibility will take place in EndNote. Two independent reviewers will screen for eligibility and inclusion in meta-analysis. Extraction will take place into a bespoke spreadsheet (Excel), which will be piloted with the reviewers prior to the study.

Information extracted will be (i) establishing eligibility in terms of dementia prevalence and age (ii) defining cardiovascular risk profile and statin treatment (iii) the control condition, and (iv) whether eligible to any subgroups or any subgroups examined. In particular, the following subgroups would be considered: by history of cardiovascular disease; by type of dementia (e.g. Alzheimer's vs vascular / mixed); by severity of dementia; and by age.

Primary outcomes will be extracted at least two years following start of observation. If multiple time-points are recorded, those at or soonest after two years will be prioritised. Both dichotomous and time-to-event (i.e. hazard ratio) data will be recorded if provided.

Outcomes:

• heart attack;

• stroke or stroke / TIA;

• TIA;

• death;

• unplanned admissions (also as number of admissions if provided).

Any compound outcome that includes the above will be extracted if the individual items are not available separately or outcomes have been combined to account for 'competing risks' – i.e. a participant who experiences death cannot experience a heart attack after this event.

Secondary outcomes will be extracted at least two years following start of observation, except for adverse drug events and drug interactions, which can be at any timepoint. If multiple time-points are recorded, those at or soonest after two years will be prioritised (including for adverse events):

• Dementia progression (any rating tool, continuous or discrete)

• Admission to care home (dichotomous or TTE)

• Adverse drug event (as defined by study)

• Drug interaction (as defined by study)

• Quality of life (any measure, EQ5D preferred if multiple)

• Falls (dichotomous, number or TTE)

• Fractures (dichotomous or TTE)

• Change in frailty (any rating tool, continuous or discrete)

• Any patient-reported outcomes

Except for adverse drug events, compound outcomes not be extracted, except where combined to account for competing risks.

**Risk of bias in individual studies**

As we will be collecting both RCT and observational studies, we will require two methods of assessing bias. For RCTs we will use the Cochrane Collaboration tool, which covers domains of: sequence generation, allocation concealment, blinding, incomplete outcome data (e.g. dropouts and withdrawals) and selective outcome reporting, which are each rated as high or low risk, or unsure. For observational studies we will use the Newcastle-Ottowa Quality Assessment Scale for case-control and cohort studies, which coves domains of: selection, comparability, exposure/outcome.

For observational studies, it is important that confounders are considered in the studies, and as part of the risk of bias assessment, the key confounder for these studies is the cardiovascular risk. At the least, we would expect for the presence of coronary / cerebral artery disease to be controlled – this would usually be specified by saying whether the aim of the statin was for primary prevention (preventing arterial disease and first heart attack / stroke) vs secondary prevention (preventing heart attacks and strokes in people who already have symptoms of coronary / cerebral artery disease or have already had a heart attack or stroke). Age and other medication to reduce vascular risk (antiplatelets and ACE-inhibitor or equivalent) will be secondary features that we would prefer to be controlled.

**Data Synthesis**

We will consider quantitative analysis including meta-analysis if studies are sufficiently similar in design and reporting. RCTs and non-RCTs will be analysed separately using a random-effects model. Relative risk will be the preferred output if available, with hazard ratio being used if presented alone, and mean difference for continuous outcomes. I2 will be used to assess any potential heterogeneity. We will consider subgroup analyses if there is sufficient suitable data to subgroup by cardiovascular risk (primary prevention vs secondary prevention), dementia severity (mild vs moderate vs severe), dementia sub-diagnosis (Alzheimer vs vascular vs Lewy Body / Parkinson vs other dementias) or age.

Evidence of publication bias will be assessed via funnel plot if data is suitable. It is considered that around ten studies are needed for a valid funnel plot.

**Confidence in cumulative evidence**

The strength of the body of evidence will be assessed for each primary and secondary outcome for which there was a data extracted by allocation of a GRADE rating of high, moderate, low or very low confidence in the result based on risk of bias, consistency, directness, precision and publication bias. High confidence means that further research is very unlikely to change our confidence in the estimate of effect, whereas low confidence indicates that it is likely that future research will have an important impact on our confidence in the estimate of effect and is likely to change the estimate.

**Discussion**

This review will look at the evidence for using aspirin for cardiovascular risk management in people who have dementia. Aspirin is a common medication prescribed for older people, and therefore is likely to be prescribed for many people with dementia. Since there is no definitive evidence that aspirin helps dementia per se, people with dementia and those that care for them need to know about risks and benefits for aspirin's primary purpose in people like them in order to make decisions for themselves.

Bishara, D. and D. Harwood (2014). "Safe prescribing of physical health medication in patients with dementia." International journal of geriatric psychiatry **29**(12): 1230-1241.

Clague, F., S. W. Mercer, G. McLean, E. Reynish and B. Guthrie (2016). "Comorbidity and polypharmacy in people with dementia: insights from a large, population-based cross-sectional analysis of primary care data." Age and ageing **46**(1): 33-39.

Eshetie, T. C., T. A. Nguyen, M. H. Gillam and L. M. Kalisch Ellett (2018). "A narrative review of problems with medicines use in people with dementia." Expert opinion on drug safety **17**(8): 825-836.

Jaturapatporn, D., M. G. E. K. N. Isaac, J. McCleery and N. Tabet (2012). "Aspirin, steroidal and non‐steroidal anti‐inflammatory drugs for the treatment of Alzheimer's disease." Cochrane Database of Systematic Reviews(2).

Mueller, C., M. Molokhia, G. Perera, N. Veronese, B. Stubbs, H. Shetty, D. Codling, J. Huntley and R. Stewart (2018). "Polypharmacy in people with dementia: Associations with adverse health outcomes." Experimental gerontology **106**: 240-245.

Rands, G., M. Orrel, A. Spector and P. Williams (2004). "Aspirin for vascular dementia (Cochrane Review)." The Cochrane Library(2).

Zhang, C., Y. Wang, D. Wang, J. Zhang and F. Zhang (2018). "NSAID Exposure and Risk of Alzheimer's Disease: An Updated Meta-Analysis From Cohort Studies." Front Aging Neurosci **10**: 83.

Zhang, H. and T. Wu (2018). A systematic review and meta-analysis of efficacy and safety of non-steroidal anti-inflammatory drugs for the treatment of Alzheimer's disease (PROSPERO protocol). PROSPERO International prospective register of systematic reviews. http://www.crd.york.ac.uk/PROSPERO/display_record.php?ID=CRD42018106730


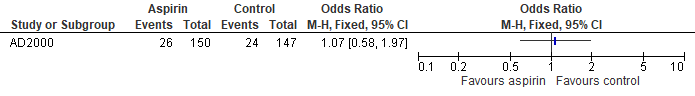


Supplementary Figure S1 Aspirin vs control: Deaths at 2y


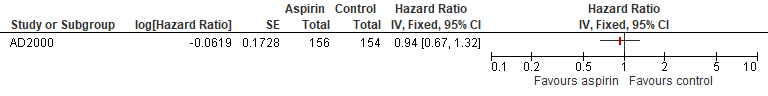


Supplementary Figure S2 Aspirin vs control: Admission to care home at 3y


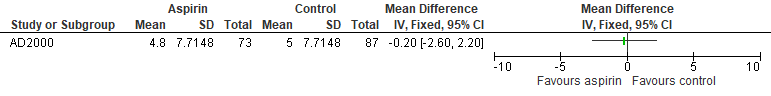


Supplementary Figure S3 Aspirin vs control: MMSE decline at 2y


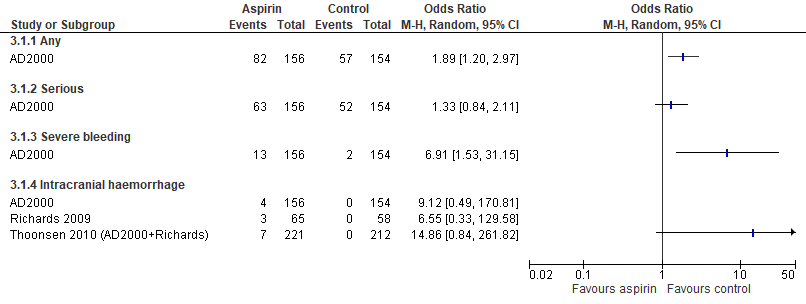


Supplementary Figure S4 Aspirin vs control: Adverse events during trial

Supplementary Table S1 (a) PRISMA harms checklist

| Section/topic (page no) | Item | PRISMA checklist item | PRISMA harms (minimum) | Recommendations for reporting harms in systematic reviews (desirable) | Check if done |
| --- | --- | --- | --- | --- | --- |
| **Title** | | | | |  |
| Title (3) | 1 | Identify the report as a systematic review, meta-analysis, or both. | Specifically mention “harms” or other related terms, or the harm of interest in the review. | — | x |
| **Abstract** | | | | |  |
| Structured summary (4) | 2 | Provide a structured summary including, as applicable: background; objectives; data sources; study eligibility criteria, participants, and interventions; study appraisal and synthesis methods; results; limitations; conclusions and implications of key findings; systematic review registration number. | — | Abstracts should report any analysis of harms undertaken in the review, if harms are a primary or secondary outcome. | x |
| **Introduction** | | | | |  |
| Rationale (5) | 3 | Describe the rationale for the review in the context of what is already known. | — | It should clearly describe in introduction or in methods section which events are considered harms and provide a clear rationale for the specific harm(s), condition(s), and patient group(s) included in the review. | x |
| Objectives (5) | 4 | Provide an explicit statement of questions being addressed with reference to participants, interventions, comparisons, outcomes, and study design (PICOS). | — | PICOS format should be specified, although in systematic reviews of harms the selection criteria for P, C, and O may be very broad (same intervention may have been used for heterogeneous indications in a diverse range of patients) | x |
| **Methods** | | | | |  |
| Protocol and registration (6) | 5 | Indicate if a review protocol exists, if and where it can be accessed (eg, web address), and, if available, provide registration information including registration number. | — | No specific additional information is required for systematic reviews of harms. | x |
| Eligibility criteria (6) | 6 | Specify study characteristics (eg, PICOS, length of follow-up) and report characteristics (eg, years considered, language, publication status) used as criteria for eligibility, giving rationale. | — | Report how handled relevant studies (based on population and intervention) when the outcomes of interest were not reported. Report choices for specific study designs and length of follow-up. | x (see ST1) |
| Information sources (7) | 7 | Describe all information sources (eg, databases with dates of coverage, contact with study authors to identify additional studies) in the search and date last searched. | — | Report if only searched for published data, or also sought data from unpublished sources, from authors, drug manufacturers and regulatory agencies. If includes unpublished data, provide the source and the process of obtaining it. | x |
| Search (7) | 8 | Present full electronic search strategy for at least one database, including any limits used, such that it could be repeated. | — | If additional searches were used specifically to identify adverse events, authors should present the full search process so it can be replicated. | protocol |
| Study selection (8) | 9 | State the process for selecting studies (ie, screening, eligibility, included in systematic review, and, if applicable, included in the meta-analysis). | — | If only included studies reporting on adverse events of interest, defined if screening was based on adverse event reporting in title/abstract or full text. If no harms reported in the text, report if any attempt was made to retrieve relevant data from authors. | x |
| Data collection process (9) | 10 | Describe method of data extraction from reports (eg, piloted forms, independently, in duplicate) and any processes for obtaining and confirming data from investigators. | — | No specific additional information is required for systematic reviews of harms. | protocol |
| Data items (9) | 11 | List and define all variables for which data were sought (eg, PICOS, funding sources) and any assumptions and simplifications made. | — | Report the definition of the harm and seriousness used by each included study (if applicable). Report if multiple events occurred in the same individuals, if this information is available. Consider if the harm may be related to factors associated with participants (eg, age, sex, use of medications) or provider (eg, years of practice, level of training). Specify if information was extracted and how it was used in subsequent results. Specify if extracted details regarding the specific methods used to capture harms (active/passive and timing of adverse event). | x |
| Risk of bias in individual studies (10) | 12 | Describe methods used for assessing risk of bias of individual studies (including specification of whether this was done at the study or outcome level), and how this information is to be used in any data synthesis. | — | The risk of bias assessment should be considered separately for outcomes of benefit and harms. | x |
| Summary measures (11) | 13 | State the principal summary measures (eg, risk ratio, difference in means). | — | No specific additional information is required for systematic reviews of harms. | x |
| Synthesis of results (11) | 14 | Describe the methods of handling data and combining results of studies, if done, including measures of consistency (eg, I^2^) for each meta-analysis. | Specify how zero events were handled, if relevant. |  | x |
| Risk of bias across studies (11) | 15 | Specify any assessment of risk of bias that may affect the cumulative evidence (eg, publication bias, selective reporting within studies). | — | Present the extent of missing information (studies without harms outcomes), any factors that may account for their absence, and whether these reasons may be related to the results. | x |
| Additional analyses (12) | 16 | Describe methods of additional analyses (eg, sensitivity or subgroup analyses, meta-regression), if done, indicating which were prespecified. | — | Sensitivity analyses may be affected by different definitions, grading, and attribution of adverse events, as adverse events are typically infrequent or reported using heterogeneous classifications. Report the number of participants and studies included in each subgroup. | x |
| **Results** | | | | |  |
| Study selection (13) | 17 | Give numbers of studies screened, assessed for eligibility, and included in the review, with reasons for exclusions at each stage, ideally with a flow diagram. | — | If a review addresses both efficacy and harms, display a flow diagram specific for each (efficacy and harm). | Only one flow-chart done. Information is available in ST2 |
| Study characteristics (14) | 18 | For each study, present characteristics for which data were extracted (eg, study size, PICOS, follow-up period) and provide the citations. | Define each harm addressed, how it was ascertained (eg, patient report, active search), and over what time period. | Add additional characteristics to: “P” (population) patient risk factors that were considered as possibly affecting the risk of the harm outcome. “I” (intervention) professional expertise/skills if relevant (for example if the intervention is a procedure). “T” (time) timing of all harms assessments and the length of follow-up. | x |
| Risk of bias within studies (15) | 19 | Present data on risk of bias of each study and, if available, any outcome level assessment (see item 12). | — | Consider the possible sources of biases that could affect the specific harm under consideration within the review. Sample selection, dropouts and measurement of adverse events should be evaluated separately from the outcomes of benefit as described in item 12, above. | x ST2 and ST3 |
| Results of individual studies (16) | 20 | For all outcomes considered (benefits or harms), present, for each study: (a) simple summary data for each intervention group (b) effect estimates and confidence intervals, ideally with a forest plot. | — | Report the actual numbers of adverse events in each study, separately for each intervention. | x ST2 and ST3 and figures SF1-4 |
| Synthesis of results (17) | 21 | Present results of each meta-analysis done, including confidence intervals and measures of consistency. | Describe any assessment of possible causality. | If included data from unpublished sources, report clearly the data source and the impact of these studies to the final systematic review. | x |
| Risk of bias across studies (18) | 22 | Present results of any assessment of risk of bias across studies (see item 15). | — | No specific additional information is required for systematic reviews of harms. See item 15 above. | x |
| Additional analysis (18) | 23 | Give results of additional analyses, if done (eg, sensitivity or subgroup analyses, meta-regression (see item 16)). | — | No specific additional information is required for systematic reviews of harms. | nil done |
| **Discussion** | | | | |  |
| Summary of evidence (18) | 24 | Summarise the main findings including the strength of evidence for each main outcome; consider their relevance to key groups (eg, healthcare providers, users, and policy makers). | — | No specific additional information is required for systematic reviews of harms. | x |
| Limitations (18) | 25 | Discuss limitations at study and outcome level (eg, risk of bias), and at review level (eg, incomplete retrieval of identified research, reporting bias). | — | Recognise possible limitations of meta-analysis for rare adverse events (ie, quality and quantity of data), issues noted previously related to collection and reporting. | x |
| Conclusions (19) | 26 | Provide a general interpretation of the results in the context of other evidence, and implications for future research. | — | State conclusions in coherence with the review findings. When adverse events were not identified we caution against the conclusion that the intervention is “safe,” when, in reality, its safety remains unknown. | x |
| **Funding** | | | | |  |
| Funding (19) | 27 | Describe sources of funding for the systematic review and other support (eg, supply of data); role of funders for the systematic review. | — | No specific additional information is required for systematic reviews of harms. | x |

Supplementary Table 1 (b)


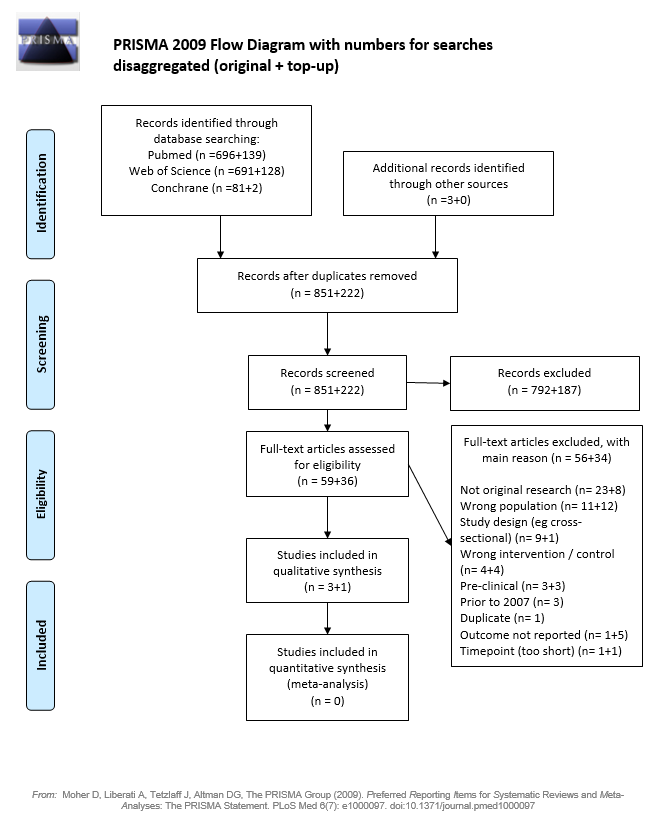


Supplementary Table S2: All studies considered at "full-text" level, and decision about inclusion status.

| **Author (year)** | **Notes** | **Included (Inc) / Excluded (Exc)** |
| --- | --- | --- |
| **Original Search** | | |
| AD2000 (2008) | Same paper as Bentham 2008 | Inc – as duplicate |
| Aisen (2008) | Commentary on AD2000 study. Reference added | Exc - not original research |
| Antithrombotic (2002) | Exc studies of ppl with dementia | Exc - not original research |
| Baskys (2012) | Review, references checked | Exc - not original research |
| Berk (2013) | Narrative review | Exc - not original research |
| Bordet (2017) | Narrative review | Exc - not original research |
| Casoli (2013) | Narrative review | Exc - not original research |
| Frautschy (2010) | Narrative review | Exc - not original research |
| Henderson (2013) | Narrative review | Exc - not original research |
| Jaturapatporn (2012) | Review, references checked | Exc - not original research* |
| Pomponi (2008) | Review of preclinical evidence | Exc - not original research |
| Pomponi (2014) | Narrative review | Exc - not original research |
| Rainsford (2007) | Narrative review | Exc - not original research |
| Rands (2012) | Review, references checked | Exc - not original research |
| Rees (2011) | Review, references checked | Exc - not original research |
| Ritter (2015) | References checked | Exc - not original research |
| Shetty (2010) | Narrative review | Exc - not original research |
| Fink (2018) | Review only looked at trials who studied people without dementia at baseline | Exc - not original research |
| Furtner (2009) | Narrative review | Exc - not original research |
| Poudel (2017) | Review of prescribing practices | Exc - not original research |
| Richard (2012) | On treatment of vascular risk factors to prevent dementia - only prevention studies references | Exc - not original research |
| Russolillo (2012) | Review of secondary prevention for stroke | Exc - not original research |
| Deschaintre (2009) | No outcome data for aspirin vs no aspirin | Exc - outcome not reported* |
| Mohammadpour (2015) | Animal models | Exc - pre-clinical |
| Nevado-Holgado (2016) | Bioinformatics study on molecular properties of drugs | Exc - pre-clinical |
| Infante-Garcia (2017) | Mouse model | Exc - pre-clinical |
| Zhou (2004) | Length six months, cognitive only | Exc - prior to 2007 (and other issues) |
| Meyer (1989) | Full text not available. Might be eligible, and useful as in vascularD if not pre 2007. Controlled trial, not randomised or blinded. | Exc - prior to 2007 (otherwise eligible for cognition)* |
| Devine (2003) | Small study of non-represent group | Exc - prior to 2007 (otherwise eligible for deaths)* |
| Bousser (2009)PERFORM | Protocol for study that excludes people with dementia | Exc - protocol only |
| Zhang (2018) | Protocol of review on NSAIDs inc Aspirin, RCTs only | Exc - protocol only |
| Bogetti (2016) | Cross-sectional | Exc - study design |
| Gorzoni (2013) | Cross-sectional | Exc - study design |
| Kimata (2008) | Exposure to aspirin is only measured on discharge, so cannot use as predictor of outcome | Exc - study design |
| McAlister (2018) | Cross-sectional data on prescribing | Exc - study design |
| Fowler (2014) | Comparison of prescribing for secondary prevention of IHD in people with and without dementia. Shows significantly less statin | Exc - study design |
| Mandas (2014b) | Cross-sectional | Exc - study design |
| Mandas (2014c) | Cross-sectional | Exc - study design |
| Mostaza (2019) | Cross-sectional data on prescribing | Exc - study design |
| Staszewski (2017) | No intervention | Exc - study design |
| Jiang (2012) | < six months, no control group | Exc - too short |
| Staszewski (2018) | Looking at different outcomes in people with aspirin resistance | Exc - wrong intervention / control |
| Taguchi (2013) | Control group discontinued due to side-effects. No correction for CVD. | Exc - wrong intervention / control |
| Yang (2017) | No control group | Exc - wrong intervention / control |
| Richard (2009) | Complex intervention rather than simply medication, unable to extract aspirin outcomes, however further results presented in Thoonsen re intracranial haem | Exc - wrong intervention / control* |
| Biffi (2010) | No dementia at b/l | Exc - wrong population |
| Chang (2016) | Outcome was notes mention of dementia | Exc - wrong population |
| Cote (2012) | No dementia at b/l | Exc - wrong population |
| Jorgensen (2011) | Only 13% have dementia. Possible could get some case-control numbers out if not enough specific | Exc - wrong population |
| Kimura (2009) | Article not in English. Unclear how many have dementia, but only 9 of 85 taking Donepezil. Case only, so probably not useful | Exc - wrong population |
| Paganini-Hill ( | Assumed no dementia at baseline | Exc - wrong population |
| Stocks (2019)ASPREE | Dementia excluded | Exc - wrong population |
| Wu (2018) | Outcome incident dementia | Exc - wrong population |
| Zhang (2014) | No dementia at b/l | Exc - wrong population |
| Rohde (2016) | Outcome was onset of CI | Exc - wrong population |
| Staszewski (2015) | Unclear the proportion who would meet criteria for dementia, and outcome is poorly defined | Exc - wrong population |
| Bentham A.D. Collaborative Group (2008) |  | Inc |
| Ferrari (2018) |  | Inc |
| Thoonsen (2010) | Presents more data on Richard 2009 and Bentham 2008 | Inc - duplicate AD2000 study |
| **Top-up search** | | |

| Laborde (2020) | Aspirin use is outcome | Exc - No suitable outcomes |
| --- | --- | --- |
| McNeil (2020) | Excludes ppl with dementia | Exc - No suitable outcomes |
| Pasina (2020) | Aspirin use is outcome | Exc - No suitable outcomes |
| Springer (2020) | Aspirin use is outcome | Exc - No suitable outcomes |
| Zupanic (2020) | Aspirin use is outcome | Exc - No suitable outcomes |
| Paseban (2019) | Animal model | Exc - Not clinical research |
| Patel (2020) | Review molecular mechanisms | Exc - Not clinical research |
| Yang (2019) | Animal model | Exc - Not clinical research |
| Al Jerdi (2020) | Review post-stroke cognition | Exc - Not original research |
| Barry (2020) | Review aspirin use | Exc - Not original research |
| Hybiak (2020) | Review aspirin applications | Exc - Not original research |
| Ji (2019) | Review prevention dementia | Exc - Not original research |
| Jordan (2020) | Review prevention dementia | Exc - Not original research |
| Narula (2019) | Review embolic disease | Exc - Not original research |
| Pinho (2019) | Review ICH | Exc - Not original research |
| Han (2019) | Cilostazol in CVD | Exc - Protocol only |
| Anastasio (2019) | Analysis of multiple medications and cognitive outcome, no comparator group | Exc - Study design |
| Wang (2019) | Acute effects post-stroke | Exc - Too short |
| Kwon (2020) | Compares aspirin and Cilostazol | Exc - Wrong comparator |
| Lee (2019) | Prevention cognitive impairment | Exc - Wrong comparison |
| McHutchinson (2020) | Review Cilostazol | Exc - Wrong intervention |
| Zeng (2019) | Review anticoagulation | Exc - Wrong intervention |
| Benito-León (2019) | Prevention of dementia | Exc - Wrong population |
| Chaturvedi (2019) | Aspirin use is outcome | Exc - Wrong population |
| Ebell (2019) | Commentary on McNeill | Exc - Wrong population |
| Guidoux (2019) | Prevention of stroke | Exc - Wrong population |
| Khezrian (2020) | Prevention cognitive impairment | Exc - Wrong population |
| Li (2019) | Outcome is dementia | Exc - Wrong population |
| Matsumoto (2020) | Outcome is dementia | Exc - Wrong population |
| Petroni (2020) | Prevention cognitive impairment | Exc - Wrong population |
| Poyuran (2019) | Case series | Exc - Wrong population |
| Rivard (2019) | Prevention cognitive impairment | Exc - Wrong population |
| Ryan (2020)* | Prevention cognitive impairment | Exc - Wrong population |
| Yang (2020) | Prevention dementia | Exc - Wrong population |
| Lee (2020) | ICH outcome | Inc |

* = mentioned in text


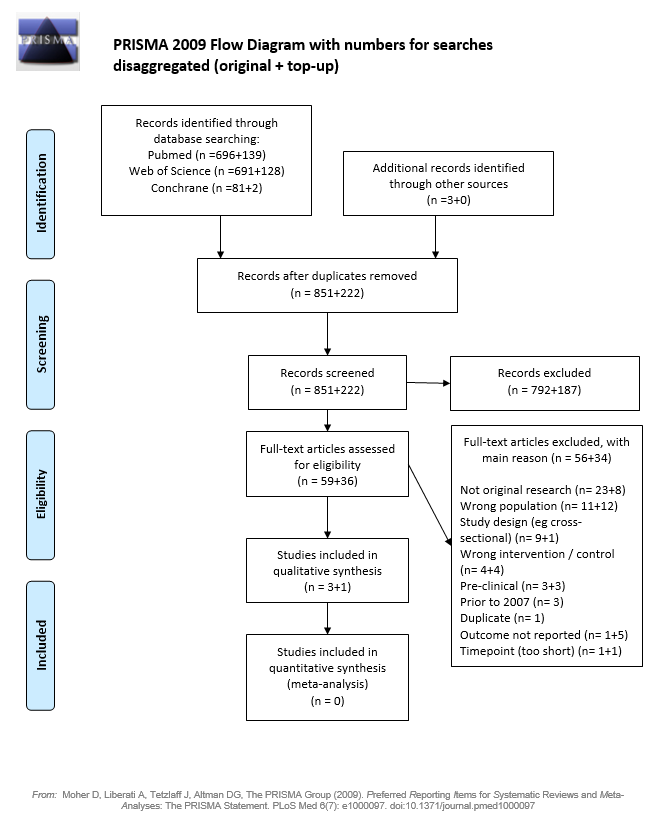


Supplementary Table S3 Risk of bias assessment for AD2000

| Study  Outcome | months | Aspirin | (%, 95%CI) | Control | (%, 95%CI) | Random. | Deviation | Missing data | Measurement | Selective | Overall risk of bias |
| --- | --- | --- | --- | --- | --- | --- | --- | --- | --- | --- | --- |
| **AD2000** |  |  |  |  |  | Minimisation technique, remotely administered, good balance achieved | Open label. Switching aspirin group > avoid aspirin group. ITT. | At 36m (aspirin n=156 / avoid n=154): Deaths 42/41, Censored 22/22, LTFU 38/50. Remaining participants: 54 (35%) aspirin / 41 (27%) avoid.  Appropriate sensitivity analyses. | Appropriate measures. Unstated if assessors blinded, may affect some outcomes | Well reported. Multiple time-points appropriately reported |  |
| *Efficacy* |  |  |  |  |  |  |  |  |  |  |  |
| Mortality | 24 | 26/150 | (17%, 12-24) | 24/147 | (16%, 11-23) | Low | High | Some concern | Low | Low | SC |
| MMSE score change | 36 | MD +0.10 | (-0.37-+0.57) | Ref |  | Low | High | Some concern | Some concern | Low | High |
| Admission to institutional care | 36 | HR 0.94 | (0.67-1.31) | Ref |  | Low | High | Some concern | Low | Low | SC |
| *Adverse events* |  |  |  |  |  |  |  |  |  |  |  |
| Any | 36 | 82/156 | (53%, 45-60) | 57/154 | (37%, 30-45) | Low | Some concern | Some concern | Some concern | Low | SC |
| Serious | 36 | 63/156 | (40%, 33-48) | 52/154 | (34%, 27-42) | Low | Some concern | Some concern | Low | Low | SC |
| Severe bleeding | 36 | 13/156 | (8%, 5-14) | 2/154 | (1%, 0-5) | Low | Some concern | Some concern | Low | Low | SC |
| Intracranial hemorrhage | 36 | 4/156 | (3%, 1-6) | 0/156 | (0%, 0-2) | Low | Some concern | Some concern | Low | Low | SC |

HR = Hazard ratio, ITT = Intention to treat analysis, LTFU = Loss to follow-up, MD = mean difference, MMSE = Mini-mental state exam, SC = Some concerns (see below)

^b^Domains of Cochrane risk of bias tool 2. Domains – Randomisation, Deviation from intended treatment, Missing outcomes data, Measurement of outcome, Selective outcome reporting – each rated “Low”, “Some concerns” (SC) or “High” risk of bias.

Supplementary Table S4 Risk of bias assessment for observational studies (Ferrari 2018 and Lee 2020)

| Study | Outcome domain | Timepoint (months) | Results summary | Selection^a^ | Comparability^a^ | Outcome^a^ |
| --- | --- | --- | --- | --- | --- | --- |
| Ferrari 2018 | Dementia progression | 24 | Rapid MMSE decline defined as faster than median decline:   - At least 2pt decline in first 12 months - and at least 4 pt decline over 24 months.   Analysis: Multinomial logistic regression on rapid decline including age, gender, education, MMSE at baseline, cerebrovascular pathology, vascular disease, family history, extrapyramidal signs.  Odds ratio rapid decline given aspirin: 0.34 (0.11-0.88) | ⌧⌧⌧🞏  Limited to those seen in neurology dept with MMSEs at least 2y apart (where convention was to carry out every six months): not clear how representative. Non-exposed from same cohort. It is not clear how aspirin use was ascertained | ⌧⌧  Study controls for vascular disease, also age, education and apoE4 status | ⌧🞏🞏  As routinely collected data as outcome, probably not blind, but objective score. Adequate follow-up, but only those with follow-up are described, so loss to follow-up unknown. |
| Lee 2020 | Intra-cranial haemorrhage | Ave 4.8y | The rate of clinical ICH in people without dementia on no antiplatelet medication weighted by propensity score was 1.20 per 1,000 person years.  ICH was more common in people with dementia, and the risk was not significantly higher for those taking aspirin (2.56 per 1,000; HR = 2.22, 1.07-4.62) than those taking no antiplatelet (2.70 per 1,000; HR = 2.02, 1.10-3.72). | ⌧⌧⌧⌧  National database offers good coverage of those who have a diagnosis, and will cover all prescribed aspirin, although some may not be prescribed. Duration of aspirin use not described, but available from authors. Those with previous ICH appropriately excluded. | ⌧🞏  Study performs matching and then weighting to control for differences between the groups, including a propensity score for being prescribed antiplatelet, and then adjusts the cox model for outstanding confounders. Includes vascular risk factors and age, but no explicit correction for other medication. | ⌧⌧⌧  By record linkage (and likely to present), adequate follow-up, analysis considered competing risk or mortality |

Key: MMSE = mini-mental state examination

^a^Domains of the risk-of-bias tool Newcastle - Ottawa quality assessment scale. Higher grade represents better more quality criteria met.
